# Perceived stress and generalized anxiety in the Indian population due to lockdown during the COVID-19 pandemic: a cross-sectional study

**DOI:** 10.12688/f1000research.26371.2

**Published:** 2021-05-05

**Authors:** Naina Wakode, Santosh Wakode, John Santoshi

**Affiliations:** 1 Department of Anatomy, ABV Government Medical College, Vidisha, MP, 464001, India; 2 Department of Physiology, All India Institute of Medical Sciences, Bhopal, MP, 462020, India; 3 Department of Orthopaedics, All India Institute of Medical Sciences, Bhopal, MP, 462020, India

**Keywords:** COVID-19, lockdown, perceived stress, anxiety, stressors, India, PSS-10, GAD-7

## Abstract

**Background: **Research on the psychosocial toll of the COVID-19 pandemic is being conducted in various countries. This study aimed to examine stress levels and causal stressors for perceived stress and generalized anxiety in the Indian population related to the lockdown during the COVID-19 pandemic.

**Methods: **A total of 300 adults were invited to participate in the online study via snowball and virtual snowball sampling. They were requested to complete electronic survey forms for assessing perceived stress and anxiety, and questions related to psychosocial stressors. Frequency and percentage were used for categorical variables. One-way ANOVA test was applied to compare responses based on gender, level of education, employment, and place of residence. A p-value of <0.05 was considered statistically significant.

**Result: **In total, 257 out of the 300 invited, responded and completed the survey. Men accounted for 58% (n=149) of the respondents. Overall, 84% (n=217) of participants had moderate to severe levels of perceived stress and 88% (n=228) had moderate to severe levels of anxiety. Women, as well as those not employed, reported significantly higher perceived stress and anxiety, urban residents reported higher perceived stress, while level of education had no difference in terms of perceived stress as well as anxiety. Fear of contracting COVID-19 was the highest stressor followed by difficulties in executing a routine exercise schedule and worry about the future.

**Conclusion: **The psychosocial impact of the nationwide lockdown on the Indian population has been high. Vulnerable groups for increased stress and anxiety include women, younger ages, and the unemployed. The stressors recognized include fear of contracting COVID-19, inability to execute a routine exercise schedule and worry about the future.

## Introduction

Since the beginning of 2020, humanity has been confronted with a pandemic caused by the severe acute respiratory syndrome coronavirus-2 that causes coronavirus disease 2019 (COVID-19) ^1^. The government of India declared a 21-day nationwide ‘lockdown’ from 25 ^th^ March 2020, which was subsequently extended in phases till 31 ^st^ May 2020, to break the cycle of spread of infection. The lockdown was in tune with the initiatives taken by many countries across the globe against this pandemic ^2, 3^.

‘Lockdown’ is an emergency protocol and is a means of preventing the public from moving from one place to the other. This led to shutting down of all activities except those considered ‘essential services’, which included healthcare, police, sanitation, grocery shops, petrol stations and fire stations. All educational institutions, offices, factories, shopping malls, religious places, and public transport, including buses, railways and aeroplanes, were completely shut down, and sports, religious ceremonies, family functions and all outdoor activities were strictly prohibited.

While isolation and lockdown are recognized as effective strategies of social distancing to stop the spread of COVID-19, the reduced access to family, friends, and other social support systems causes loneliness, increasing mental health issues like anxiety and depression ^4– 6^.

Researchers, in the past and during this present crisis, have tried to address the psychological stress in healthcare providers ^7– 9^ and the general population ^2, 3, 10^. The present study, conducted during the fourth phase of nationwide lockdown, from 18 ^th^ to 31 ^st^ May 2020, attempts to examine levels of perceived stress and generalized anxiety disorders and causal stressors among the Indian population related to the COVID-19 pandemic and consequent lockdown.

## Methods

### Study design and participants

We conducted an online survey wherein 300 participants were invited via snowball and virtual snowball sampling; the sample size was decided on the basis of logistics and time availability for the study. The study was approved by the Institutional Ethics Committee of the ABV Government Medical College, Vidisha, MP, India (reference no. 21(b)/IEC/ABV GMC/Vidisha/2020).

A link to the electronic survey forms ( *Extended* data ^11^) was posted on Facebook, and was sent via WhatsApp by the authors to multiple contacts, including colleagues and acquaintances that were from a wide section of society. Consent to participate was implied if the participant completed the questionnaire. The items for the questionnaire were derived from previous study on the topic ^12^.

Inclusion criteria of participants were: a) aged >18 years; b) have an internet connection and Facebook or WhatsApp installed on their mobile phone. Those unwilling to participate or did not provide consent and those <18 years of age were excluded because the psychometric measures utilized in the study were designed for adults only.

### Data collection and survey

Data was collected from 18 ^th^ to 25 ^th^ May 2020.

The survey questionnaire, based on the perceived stress scale (PSS-10) ^12^ and Generalized Anxiety Disorder (GAD-7) ^7^ instruments, explored the psychosocial stressors among the respondents. For each potential stressor, the frequency of occurrence was classified as never, almost never, sometimes, often, and very often, and these were scored as 0, 1, 2, 3 and 4, respectively. While PSS measures perception of stress over the last month GAD measures anxiety for the last 2 weeks; both these instruments have been used in previous studies on this subject ^7, 10, 12^.

### Data analysis

The data collected were tabulated and analysed using Microsoft Excel 2016 with data analysis add-in. Frequency and percentage were used for categorical variables. One-way ANOVA test was applied to compare responses based on gender, age, level of education, and place of residence. A p-value of <0.05 was considered statistically significant.

We used the STROBE cross sectional checklist when writing our report ^13^.

## Results

**Figure 1.**
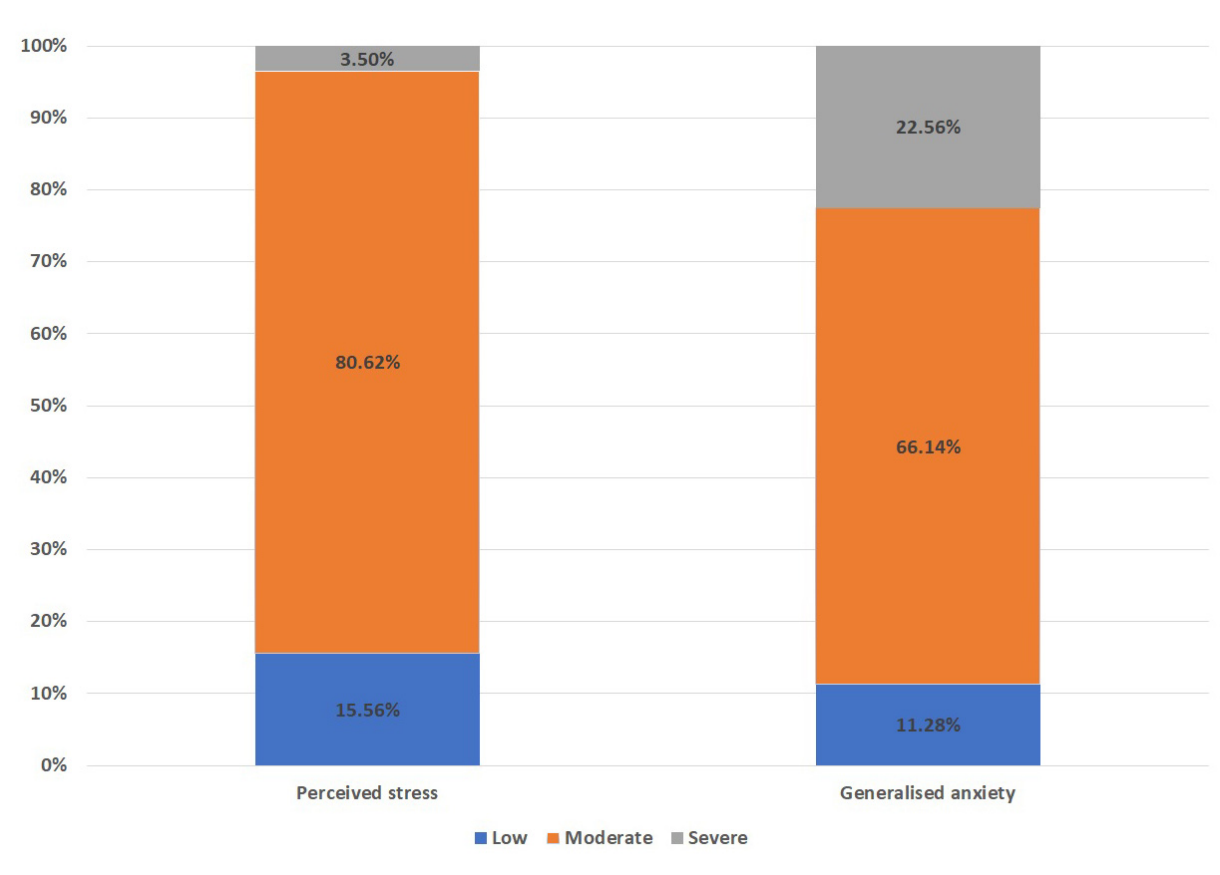
Percentage of perceived stress & generalized anxiety stress in Indian population.

A total of 257, out of the 300 participants who were sent the survey, responded and completed the survey. They belonged to central, north and western India. The mean age of the participants was 25 years. Men constituted 58% (n=149) of the respondents. Overall, 84% (n=217) of participants had moderate to severe levels of perceived stress and 88% (n=228) had moderate to severe levels of anxiety ( Figure 1).

Table 1 shows the PSS-10 and GAD-7 scores of the study participants as stratified by gender, age, level of education, and place of residence. Women as well as those not employed reported significantly higher perceived stress and anxiety, urban residents reported higher perceived stress while the level of education had no difference in terms of perceived stress as well as anxiety. The psychosocial impact of the lockdown due to the COVID-19 pandemic is shown in Table 2. Fear of contracting COVID-19 was the highest stressor followed by difficulties in executing routine exercise schedule and worry about the future.

**Table 1.** Demographic characteristics of participants showing perceived stress score and generalized anxiety disorder scale score.

Characteristics		N (%)	Perceived Stress Scale (PSS-10)		Generalised Anxiety Disorder (GAD-7)	
			Score	p –value	Score	p –value
Gender	Male	149 (58)	17.28±5.25	0.0028	11.92±3.974	0.01
	Female	108 (42)	19.11±4.44		12.93±2.80	
Employed	No	176 (68.48)	18.83±4.32	0.0009	13.18±2.93	0.0000006
	Yes	81 (31.51)	16.34±5.91		10.53±4.10	
Education	University	220 (85.6)	18.19±5.03	0.24	12.46±3.59	0.19
	School	37 (14.39)	17.18±4.818		11.67±3.31	
Place of residence	Urban	178 (69.26)	18.76±4.69	0.001	12.26±3.50	0.57
	Rural	79 (30.73)	16.44±5.33		12.54±3.70	

**Table 2.** Psychosocial impact of COVID-19 (rated on a Likert scale).

Statements	Frequency of occurrence (N)		
	Never / almost never	Sometimes	Often / very often
How often do you face financial strain during the lockdown period?	110	84	63
How often do you worry about the future?	81	94	82
How often do you fear contracting COVID-19?	35	57	165
How often do you feel stress due to inability to socialize?	123	81	52
How often do you face difficulties in executing your routine exercise schedule during the lockdown period?	100	72	85
How often do you face sleeping difficulties during the lockdown period?	165	48	44

## Discussion

The levels of stress and anxiety reported in the present study are similar to those reported by researchers from other countries ^2, 3, 5, 14^. The present study is in agreement with previous studies from other parts of the world where women and those with lower incomes are prone to higher levels of stress and anxiety ^2, 3, 5, 15, 16^; this was in contrast to a study from Pakistan where men reported a higher degree of stress during the current crisis ^17^. This could be attributed to cultural factors, which need further evaluation for clearer understanding.

In the present study, older respondents reported lower levels of stress. This could suggest the struggle and hardships of daily life which the younger generation is under ^18^; also, the younger generation tends to obtain a large amount of information from social media, which can easily trigger stress ^3, 10^. We found significant difference in the levels of perceived stress reported between urban and rural residents, while no such difference was noted in generalised anxiety scores.

In the present study, we found no difference in the levels of stress when considering the level of education of the respondents. Vallejo *et al.* ^19^ found those with a lower level of education to be reporting higher stress. Other studies found that those who were highly educated had a higher risk of depression; it is presumed that highly educated and professional people are forced to stay at home and delve into other aspects of family life leading to higher levels of perceived stress ^5, 10^.

When considering the psychosocial impact of COVID-19, fear of contracting COVID-19 was the highest stressor, which was consistent with other studies ^17, 20^. This was followed by difficulties in executing your routine exercise schedule and worry about the future ( Table 2).

### Limitations

This being a cross-sectional study, the selection of participants was non-random, and it is impossible to make unbiased estimates from snowball samples so the results of this study need to be interpreted with due caution. However, this was the best available method of data collection in the current circumstances. The study was also limited by the lack of other socio-demographic and cross-cultural comparison groups.

## Conclusions

The psychosocial impact of the nationwide lockdown on the Indian population has been high. The vulnerable groups for stress and anxiety include women, those of a younger age, and the unemployed. The stressors recognized include fear of contracting COVID-19, inability to execute routine exercise schedule and worry about the future.

## Data availability

### Underlying data

Figshare: Raw data PSS_GAD Psychosocial impact of lockdown.csv, https://doi.org/10.6084/m9.figshare.12860060.v2 ^11^.

### Extended data

Figshare: Raw data PSS_GAD Psychosocial impact of lockdown.csv, https://doi.org/10.6084/m9.figshare.12860060.v2 ^11^.

This project contains the following extended data:

- Online questionnaire.

Data are available under the terms of the Creative Commons Zero "No rights reserved" data waiver (CC0 1.0 Public domain dedication).
