# Supplementary material for: Perceived stress and generalized anxiety in the Indian population due to lockdown during the COVID-19 pandemic: a cross-sectional study
Source: F1000Res. 2021 Aug 4;9:1233. Originally published 2020 Oct 14. [Version 3] doi: 10.12688/f1000research.26371.3 (PMC8311798; doi:10.12688/f1000research.26371.3)
Supplement: Peer review history

# Peer review: Reviewer response for version 3

**DOI:** 10.5256/f1000research.58977.r91182

**Published:** 2021-08-13

**Authors:** Javier Santabárbara

**Affiliations:** 1 Ministry of Science and Innovation, Centro de Investigación Biomédica en Red de Salud Mental (CIBERSAM), Madrid, Spain; 2 University of Zaragoza, Zaragoza, Spain

The authors have addressed succesfully all my concerns.

Is the work clearly and accurately presented and does it cite the current literature?

Yes

If applicable, is the statistical analysis and its interpretation appropriate?

Yes

Are all the source data underlying the results available to ensure full reproducibility?

Yes

Is the study design appropriate and is the work technically sound?

Yes

Are the conclusions drawn adequately supported by the results?

Yes

Are sufficient details of methods and analysis provided to allow replication by others?

Yes

Reviewer Expertise:

Biostatistics, Epidemiology, Mental health

I confirm that I have read this submission and believe that I have an appropriate level of expertise to confirm that it is of an acceptable scientific standard.

# Peer review: Reviewer response for version 2

**DOI:** 10.5256/f1000research.56132.r89031

**Published:** 2021-07-20

**Authors:** Kiran Chaudhari

**Affiliations:** 1 Department of Pharmacology and Neuroscience, University of North Texas Health Science Center, Fort Worth, TX, USA

The authors have studied a critical and timely topic of the impact of the COVID-19 pandemic on psychosocial behavior in the Indian sub-population. The study included a simple cross-sectional design and subjective survey-based assessment using online tools. The data presented is easy to understand and in a self-explanatory fashion.

 There are some minor comments to be addressed:

- The authors mentioned that they used One-Way ANOVA for statistical analysis. It is unclear what component of the data was analyzed with the One-Way ANOVA (and any posthoc pairwise comparison test). There can be multiple ways the data is analyzed, and the simplest would be the use of the Student t-test (unpaired-test) to compare between 2 groups.
- Although the Figures and Tables are clear to understand, they need legends/footnotes with information such as a brief take-home message, what type of statistical analysis was used for that particular figure or table, and the set criteria for significance?
- It would be helpful to mention if the sample was limited to a particular region or a nationwide sample to conclude that the outcome is limited to the region or it can be applied to the whole population.

Is the work clearly and accurately presented and does it cite the current literature?

Yes

If applicable, is the statistical analysis and its interpretation appropriate?

Partly

Are all the source data underlying the results available to ensure full reproducibility?

Yes

Is the study design appropriate and is the work technically sound?

Yes

Are the conclusions drawn adequately supported by the results?

Yes

Are sufficient details of methods and analysis provided to allow replication by others?

Yes

Reviewer Expertise:

Neuroscience, cognition, anxiety, aging

I confirm that I have read this submission and believe that I have an appropriate level of expertise to confirm that it is of an acceptable scientific standard.

# Response

**Published:** 2021-07-23

**Authors:** Santosh Wakode

**Affiliations:** All India Institute of Medical Sciences, Bhopal, India

We thank the reviewer for taking out time to review our article and the comments and suggestions.

 These have definitely helped in making the article better.

- Unpaired t-test has been used for analysing the data.
- Legend of the figure has been modified for a clearer understanding
  .
- The study participants belonged to central, north, and western India.

# Peer review: Reviewer response for version 2

**DOI:** 10.5256/f1000research.56132.r89032

**Published:** 2021-07-15

**Authors:** Javier Santabárbara

**Affiliations:** 1 Ministry of Science and Innovation, Centro de Investigación Biomédica en Red de Salud Mental (CIBERSAM), Madrid, Spain; 2 University of Zaragoza, Zaragoza, Spain

This is a good article and well written. In brief, objective and robust manuscript, current references, rigid and reliable design method, results and discussion are supported by the data and conclusion responds to the object of investigation.

 This work represents an important area of inquiry that is relevant to the readership of this journal. However, additional details are needed for optimal review.

  

- The authors have obviated meta-analysis of anxiety in the general population (Santabárbara *et al. *(2020 ^1^)) and in specific populations like health workers (Santabárbara *et al. *(2021 ^2^)) during COVID-19 outbreak that should be included in the introduction and discussion sections. In addition, a recent meta-analysis reported prevalence of anxiety in South Asia and also should be discussed (Hossain *et al. *(2021 ^3^)).
- Data analysis: Please, replace ANOVA by t-test.
- Table 2: Please, add percentages and chi-square test p-value to compare the categories.

Is the work clearly and accurately presented and does it cite the current literature?

Yes

If applicable, is the statistical analysis and its interpretation appropriate?

Yes

Are all the source data underlying the results available to ensure full reproducibility?

Yes

Is the study design appropriate and is the work technically sound?

Yes

Are the conclusions drawn adequately supported by the results?

Yes

Are sufficient details of methods and analysis provided to allow replication by others?

Yes

Reviewer Expertise:

Biostatistics, Epidemiology, Mental health

I confirm that I have read this submission and believe that I have an appropriate level of expertise to confirm that it is of an acceptable scientific standard, however I have significant reservations, as outlined above.

## References

[1] : Prevalence of anxiety in the COVID-19 pandemic: An updated meta-analysis of community-based studies. *Progress in Neuro-Psychopharmacology and Biological Psychiatry*.2021;109: 10.1016/j.pnpbp.2020.11020710.1016/j.pnpbp.2020.110207PMC783465033338558

[2] : Prevalence of anxiety in health care professionals during the COVID-19 pandemic: A rapid systematic review (on published articles in Medline) with meta-analysis. *Progress in Neuro-Psychopharmacology and Biological Psychiatry*.2021;107: 10.1016/j.pnpbp.2021.11024410.1016/j.pnpbp.2021.110244PMC918843233453320

[3] : Prevalence of anxiety and depression in South Asia during COVID-19: A systematic review and meta-analysis.*Heliyon*.2021;7(4) : 10.1016/j.heliyon.2021.e06677e0667710.1016/j.heliyon.2021.e0667733898819PMC8056240

# Response

**Published:** 2021-07-23

**Authors:** Santosh Wakode

**Affiliations:** All India Institute of Medical Sciences, Bhopal, India

We thank the reviewer for taking out time and reviewing our article.

 Unpaired t-test has been used to analyse the data.

 We thank the reviewer for suggesting the articles for discussion.

# Peer review: Reviewer response for version 1

**DOI:** 10.5256/f1000research.29113.r79580

**Published:** 2021-03-10

**Authors:** Krishna Prasad Muliyala

**Affiliations:** 1 Department of Psychiatry, National Institute of Mental Health and Neuro Sciences (INI), Bangalore, Karnataka, India

Please mention what GAD measures under the section of data collection and survey. Whereas, PSS measures perception of stress over the last month. GAD measures anxiety for the last 2 weeks.

 What was the mean age of the respondents?

 How were the items for the questions in Table 2 selected?

 What is the time frame for these questions?

 Which parts of India did the respondents belong to? The positivity rates and lockdowns may have had differential impact.

Is the work clearly and accurately presented and does it cite the current literature?

Yes

If applicable, is the statistical analysis and its interpretation appropriate?

Yes

Are all the source data underlying the results available to ensure full reproducibility?

Yes

Is the study design appropriate and is the work technically sound?

Yes

Are the conclusions drawn adequately supported by the results?

Yes

Are sufficient details of methods and analysis provided to allow replication by others?

Partly

Reviewer Expertise:

Psychiatry, mental health

I confirm that I have read this submission and believe that I have an appropriate level of expertise to confirm that it is of an acceptable scientific standard.

# Response

**Published:** 2021-04-13

**Authors:** Santosh Wakode

**Affiliations:** All India Institute of Medical Sciences, Bhopal, India

We thank the reviewer for the valuable comments.

 GAD measures anxiety for the past 2 weeks.

 The mean age of the respondents was 25 years.

 The items for the questions in Table 2 were selected based on previous studies on this subject. (reference 12).

 The time frame for the questions was the first week of 4 ^th^ phase of nationwide lock down.

 The respondents were mainly from central, north and western India.
